# Effect of organic biostimulants on cannabis productivity and soil microbial activity under outdoor conditions

**DOI:** 10.1186/s42238-024-00214-2

**Published:** 2024-03-26

**Authors:** Jose F. Da Cunha Leme Filho, Bee K. Chim, Cameron Bermand, Andre A. Diatta, Wade E. Thomason

**Affiliations:** 1 School of Forestry and Horticulture / School of Biological Sciences, Southern Illinois University, Carbondale, USA; 2 School of Food and Agriculture – Cooperative Extension, The University of Maine, Presque Isle, Presque Isle, USA; 3 School of Plant & Environmental Sciences, Virginia Polytechnic Institute & State University, Blacksburg, USA; 4 Department of Agronomy, Gaston Berger University, Saint-Louis, Senegal; 5 Plant and Soil Sciences, Oklahoma State University, Stillwater, USA

**Keywords:** Biofertilizer, Bioinoculant, Humic acid, Hemp, Manure tea, Soil respiration

## Abstract

In 2019 and 2020, we investigated the individual and combined effects of two biofertilizers (manure tea and bioinoculant) and one humic acid (HA) product on cannabis biochemical and physiological parameters and soil CO_2_ evolution under outdoor conditions. Our hypothesis was that HA would increase the microbial activity in the biofertilizers and synergy of both compounds would promote better plant performance and stimulate soil microbial activity. In 2020, the individual and combined application of biofertilizers and HA increased cannabis height, chlorophyll content, photosynthetic efficiency, aboveground biomass, and bucked biomass by 105, 52, 43, 122, and 117%, respectively. Impacts were greater under suboptimal growing conditions caused by planting delay experienced in 2020. In 2019, planting date occurred in-between the most favorable period and chlorophyll content and photosynthetic efficiency were the only parameters influenced by the application of biostimulants. The discrepancies between the two growing seasons reinforce the evidence of other studies that biostimulants efficacy is maximized under stress conditions. This study could not conclusively confirm that the combined use of biofertilizer + HA is a superior practice since affected plant parameters did not differ from application of the compounds singly. Similarly, only one biofertilizer + HA treatment increased soil microbial activity. More research is needed to define optimum rates and combinations of biofertilizer and stimulants for cannabis.

## Significance to the *Journal of Cannabis Research*

Cannabis (*Cannabis sativa* L.) cultivation has increased recently due to changes in legislation and regulatory protocols. Research and production recommendations are rapidly needed to support this fast-growing market. Few commercial agrochemical products are currently authorized for use in cannabis. However, biostimulants, being approved by organic certification, can be an alternative to boost productivity of cannabis varieties used for cannabinoids production.

## Introduction

The term cannabis is associated with two genetically different biotypes known as industrial hemp and marijuana (Small 2016). According to the regulations in most countries, the delta-9-tetrahydrocannabinol (THC) levels is the baseline to differentiate between biotypes, where 0.3% of THC or lower characterizes industrial hemp and marijuana is in any range above this threshold (Small and Cronquist 1976). Therefore, “*cannabis*” refers to a taxonomic genus, and before non-italicized, “cannabis” is a generic term that can compass the species diversity (Cherney and Small 2016). Narcotics regulations enacted after the Second World War resulted in drastically decreased cannabis cultivation (Callaway 2004), and consequently, scientific research, environmental impacts, and legal human experience also faded with time (Eisenstein 2015). Knowledge regarding cannabis water use, fertilizer and disease control requirement, cropping systems, and yields are outdated due to the legal status (Butsic and Brenner 2016).

Recently, interest in cannabis has resurged because of its potential as a multi-purpose crop (Fike 2016) and potential profitability (Fortenbery and Bennett 2004). Cannabis is a versatile plant that can be grown for fiber, seed, or oil (Kaiser et al. 2015), fuel (Finnan and Styles 2013), and pharmaceutical properties (Zuardi 2006). Particularly, the cannabidiol (CBD) industry is evaluating this cannabinoid as a treatment for epileptic seizures (Detyniecki and Hirsch 2015), pain (Jensen et al. 2015), and anxiety (Hagerty et al. 2015). The desire to produce cannabis secondary metabolites including cannabinoids, terpenes, and flavonoids (ElSohly and Gul 2014; Gorelick and Bernstein 2017; Hanuš et al. 2016) increases the need for research addressing ways to maximize cannabis development and yield. Plant biostimulants may help cannabis producers increase crop yield and quality (Lyu et al. 2019). Biostimulant products are commonly used in other agricultural crops and are derived from a number of biological or organic sources (Calvo et al. 2014). Biofertilizers and humic acid (HA) are under the overall category of “biostimulants” and these products promote plant growth through nutrient mobilization, hormone production, disease control, and improved stress tolerance (Brown and Saa 2015; du Jardin 2015; Kauffman et al. 2007). Some of the positive outcomes of biostimulant products might be interchangeable; for instance, more phytohormones production can enhance drought stress tolerance (EL Sabagh et al. 2022; Ullah et al. 2018). Considering the benefits previously cited, the positive effects of biostimulants on soil structure, root development, and nutrient uptake might contribute to higher productivity on cannabis.

Humic acid (HA) is an organic biostimulant compound known to increase overall plant growth (El-Ghamry et al. 2009; Peña-Méndez et al. 2005), root growth, and nutrient uptake (Tattini et al. 1990). Biofertilizers can be used to increase plant nutrient availability, crop growth and yield, and minimize negative environmental impacts (Singh et al. 2016). Compost and manure teas are example of biofertilizers (Laila et al. 2015; Ronga et al. 2018), and bioinoculants composed of many different microorganisms (Fuentes-Ramirez and Caballero-Mellado 2006) are among the biofertilizer products. There is substantial work demonstrating the positive effects of biostimulants on biomass production of many crop species (Chen and Aviad 1990; Mabood et al. 2014), and research addressing the response on cannabis is still developing. There are far less studies testing the use of biostimulants on cannabis when compared to other plants. Humic acid application on cannabis presented positive results in terms of canopy uniformity (Bernstein et al. 2019) and higher plant height, chlorophyll content, and photosynthetic efficiency (Da Cunha Leme Filho et al. 2020a, b). According to Turner et al. (1978), cannabinoid compounds are present in all aerial parts of the cannabis plant, and thus, the research to validate the responses of cannabis to the application of biostimulants can be very important because biomass increase can potentially lead to higher cannabinoids production, which is the most valuable sub-product. More biomass can contribute to improved performance overall or yield.

The addition of plant biostimulants, especially humic acid products, is often aimed at improving the nutritional status of the plants. There is a growing body of information available regarding the effects of nutrients on the cannabis plant growth and secondary metabolite production. The literature shows that cannabis plants presenting the highest cannabinoid levels were obtained under N, P, and K deficiency (Saloner and Bernstein 2021, 2022; Shiponi and Bernstein 2021). The potential cause of this trend is consistent with a yield dilution effect in the final analysis when more biomass decreases the cannabinoid concentration (Shiponi and Bernstein 2021). Furthermore, the optimal application dose of organic fertilizer (Caplan et al. 2017), NO_3_/NH_4_ supply (Saloner and Bernstein 2020), and NPK ratios (Bevan et al. 2021) are highly impacting cannabis development and secondary metabolite profile. Thus, it is crucial to learn more about how plant biostimulants interact with nutrients so that production will likely be affected.

Studies have shown that HA stimulates microbial activity as an important ion exchange and metal complexing systems using its chelating properties (Puglisi et al. 2009). Also, it increases the production of micelium by mycorrhizal fungus (Gryndler et al. 2005). Therefore, we hypothesize that when applied in combination, the HA will increase microbial activity of the microorganisms carried in biofertilizers, improving the overall performance on plant and soil parameters. In order to evaluate this premise, we assessed biochemical and physiological responses of cannabis and soil CO_2_ evolution to the combined and individual application of HA, manure tea and a bioinoculant under field conditions.

## Materials and methods

### Design of experiment and management

Field trails were conducted at Urban Horticulture Center (UHC) of Virginia Tech in 2019 and a private farm located near Blacksburg VA in 2020. The two locations presented similar soil characteristics: Silt loam, pH ranging from 6.2 to 6.5 in the soil surface and 2–6% slope. Planting date for the first growing season was July, 03, 2019, but due to delayed transplant availability associated with the COVID-19 pandemic, planting date in the second growing season was August, 01, 2020. The transplants were originated from cuttings of monoecious plants (all females). Twenty-one-day-old transplants of the variety Hawaiian Haze were planted in 1-m wide white plastic strips with drip irrigation installed underneath the plastic. Hawaiian Haze is a variety used mainly for CBD production due to its high CBD and low THC ratio. The space between plants was 1.5 m with 1.8-m row spacing for a total of 3590 plants per ha. Three biostimulant products from different resources did not contain any significant amount of plant macro or micronutrients and were applied in six treatments including control (no biostimulants). The humic acid product was MicroLife Humic Acid Complex® and the two biofertilizers were Microgeo® and Microgro Supreme Bioinoculant®. The MicroLife Humic Acid Complex® is constituted of 2% humic acid / 1% organic carbon and 15% humic acid /1% fulvic acid. One of the two biological fertilizers was Microgeo® which is a Brazilian patented product categorized as a manure tea. This biofertilizer is composed of organic compounds, active and dormant cells from various microorganisms (bacteria, yeasts, filamentous fungi, and algae), metabolites and organo-mineral chelates and it is produced through continuous anaerobic fermentation in a liquid media (D’andrea 2002). According to the technical manual, the preparation is using the CLC® (Continuous Liquid Composting) process, where 5% of the commercial biological fertilizer Microgeo ®, 15% of ruminal content and water are mixed in a tank exposed to sunlight. After 15 days, the biofertilizer is ready to be applied. The Microgro Supreme Bioinoculant® is a water-soluble powder containing 76 strains of bacteria and fungi including 11 Mycorrhizal species and microbial food (sugars, humic acid, kelp, amino acids, and yeast extract). Detailed product descriptions are provided in Table 1. All three biostimulant products did not present any significant nutrient content.

**Table 1:** Products components and full description

General Definition	Category	Subcategory	Name	Components
Biostimulants	Humic	Fulvic	Microlife Humic Acid Complex®	15% humic acid and 1% fulvic acid derived from leonardite
	Biofertilizers	Manure tea	Microgeo®	Recancitrant substances, biodynamic preparations, pentoses, minerals and brans and the microorganisms produced in the manure tea fermentation
		Bioinoculant	Microgro Supreme Bioinoculant®	76 different strains of bacteria and fungi planced on dry milk carrier loaded with microbial food. The microorganisms included are species of genus *Bacillus*, *Pseudomonas*, *Streptomycetes*, *Trichoderma*, and endo and ectomycorrhizal fungi

Preplant fertilization followed the Virginia Tech Extension tomato production guide for nutrient requirement and soil pH (O’Dell et al. 1989). Biostimulants were the only products to be applied after preplant fertilizer. Pesticides were not applied, and weeds were mechanically controlled. The experiment employed a randomized complete block design with 6 treatments and four replications with a plot size of 1.8 m × 9.1 m. The experimental unit was 5 plants/plot and the application rates of the HA and biofertilizer compounds were consistent with the label recommendation of each product. A detailed description of the treatments and application rate is shown in Table 2. Biostimulant applications were conducted as one drench application to the base of the transplants at 5 leaf pair (compound) (Mediavilla et al. 1998) while they were being established in the soil. A second drench application occurred at 8 leaf pair (compound) growth stage. A third and fourth application was performed as foliar treatments at 13 and 15 leaf pair (compound), respectively.

**Table 2:** Treatments description and application rate

No.	Treatments 2019–2020				
	Category	Product name and abbreviation	Label	Drench (ml/plant)	Foliar (ml/plot)
1		Control (C)	0	0	0
2	Humic acid	Microlife Humic (LH)	14 L/ha^a^	65	390
3	Biofertilizer	Microgeo (M)	150 L/ha	41	249^b^
4		Microgro Supreme Bio (B)	6.1 kg/ha^a^	65	390
5	Humic + biofertilizer	Microlife Humic + Microgeo (LH + M)	14 L/ha and 150 L/ha	65 and 41	390 and 249
6		Microlife Humic + Microgro Supreme Bio (LH + B)	14 L/ha and 6.1 kg/ha	65 and 65	390 and 390

^a^Microlife Humic and Microgro Supreme Bioinoculant are diluted in 234 L of water per ha

^b^Microgeo foliar applications were performed using 3% strength of the product or 4.5 L diluted in 150 L of water per ha

### Data collection

We measured cannabis height at the tallest shoot apex, photosynthetic efficiency/OS-50II fluorometer (Opti-Sciences, Tyngsboro, MA), atLEAF chlorophyll meter value (FT Green LLC, Wilmington, DE), greenness (ranked from 0 to 10, personal visual evaluation where 0 was less green and 10 was darker green) and vigor (visual assessment from 0 to 10, with 0 showing extreme poor vigor and 10 indicating greatest vigor). Vigor ratings were defined based on visual growth rate, steam diameter and leaf size. The measurements were collected from the latest fully developed leaf at the growth stages corresponded to 13 and 17 leaf pair (compound) and 2 weeks post flowering. The conversion of atLEAF units to chlorophyll content in mg/cm^2^ was performed using the tool provided in the device website (atleaf.com). At maturity all aboveground biomass was clipped, and plot weights recorded. The harvest area was 16.3 m^2^ per plot. Dry weight was measured after 20 days drying in a barn at ambient conditions. The drying period also allowed us to measure the cannabis bucked biomass which is the total biomass minus the stem. The flowers and leaves were manually separated from the stem and the bucked biomass material was weighed. After all the plants were harvested, a representative soil sample (3 cores at 10 cm depth) was collected from each plot for soil CO_2_ evolution analysis.

### Soil CO_2_ evolution

Soil samples were air dried for 2 weeks, then ground to pass through a 2-mm sieve. Soil pore space, particle density, and bulk density were determined via displacement in a solution of 0.05 molar Calgon (sodium hexametaphosphate). The soil pore space in a disturbed samples was estimated following the techniques by Franzluebbers (2016) and Franzluebbers et al. (2000), where the soil was gently compacted in graduated bottles and water added to fill 50% of the available soil porosity, assuming a particle density of 2.65 Mg m^−3^. The volume of water used to re-wet soil samples was equivalent to 50% total porosity. The 236-ml mason jars were used as the incubation vessel. Lids were modified to include a self-sealing injection port to allow for gas sampling, while maintaining the conditions of incubation. Each jar was filled with 50 g of sieved, air-dried soil (Franzluebbers 2018). The sealed lids were installed, and the jars were flushed of CO_2_ using a canister of CO_2_-free air. After flushing for 3 min, 12 ml of deionized water was injected via syringe through the self-sealing injection port.

Re-wetted soils were placed into a Fisher Scientific Isotemp Incubator at 25℃ for 72 h (Franzluebbers et al. 2000). After 3 days, these samples were removed from the incubator, and a 5 ml gas sample was collected from the headspace using a 10-ml gas sampling syringe. This sample was injected into a LI-COR Soil Gas Flux System (IRGA/infra-red gas analyzer) (Haney et al. 2008). The data recorded was compared to a calibration of known standard samples to determine CO_2_ concentration in the headspace of the incubation jar. This was reported as ug CO_2_ produced/g soil. All soil samples were run in duplicate.

### Data analysis

The univariate distribution for each variable was determined with outliers evaluated and removed when studentized residual was greater than 2.5. Statistical analyses were performed using the GLM procedure in SAS 9.4 (SAS Institute 2013) with all variables except replication considered fixed effects. Treatment effects on cannabis height, photosynthetic efficiency, atLEAF chlorophyll meter, greenness, vigor, aboveground biomass, bucked biomass, and soil CO_2_ evolution were assessed. Mean separations were performed using the Tukey-Kramer command within the LSMEANS statement when F-tests indicated that significant differences existed (*p* < 0.05) for all plant parameters and (*p* < 0.1) for soil CO_2_ evolution.

## Results and discussion

### Cannabis height

In 2019, we found no significant differences in cannabis height at any of the three data collection periods. Similarly, there are studies showing limited or no responses in terms of plant height when applying biofertilizer on chili pepper (*Capsicum chinense*) (Moreno-Salazar et al. 2020) and rice (*Oryza sativa* L.) (Naher et al. 2016) and HA on wheat (*Triticum aestivum*) (Ulukan 2008). Average height at the latest measurement was 95.4 cm in 2019 and 102.2 cm in 2020 despite the different planting dates, however a greater degree of branching was visually noted in 2019.

**Fig. 1:**
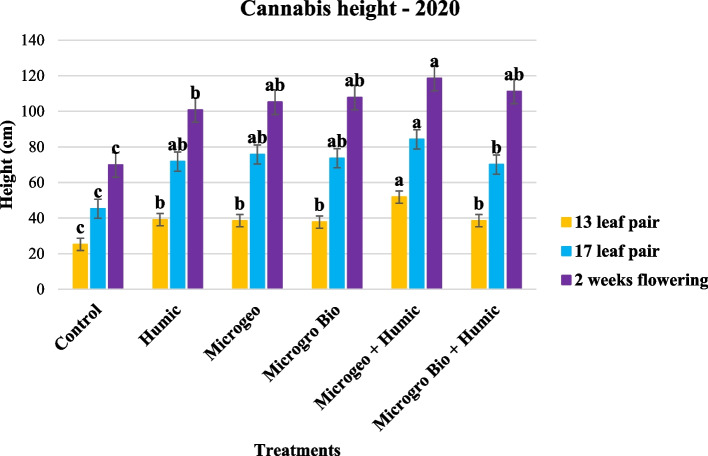
Cannabis height readings collected in 2020 at three growing stages (13, 17, and 2 weeks post flowering). Mean within a column followed the same letter by the same growth stage is not significantly different at the 0.05 probability level

In 2020, plant height was lowest at all measurement times for the control which did not receive any biostimulant product (Fig. 1). One treatment combining HA and biofertilizer products (Humic + Microgeo) generally had the greatest cannabis height compared to control and the other treatments receiving one or more biostmulant products. Biostmulant treatments receiving one or more products were taller than the control for every individual comparison or data collection period. As studies have shown that biostmulants can be more effective when plants are under stress (Bulgari et al. 2019; Romero et al. 2014), the reduction of solar illumination due the late planting could be a causative stress promoter resulting in higher biostimulant effectiveness in 2020. The application of HA compounds has increased plant height of as canola (*Brassica napus* L.) (Sani 2014), wheat (Tahir et al. 2011), and cotton (*Gossypium hirsutum* L.) (Basbag 2008). Similarly, the use of biofertilizer increased plant height on pomegranate (*Punica granatum* L.) (Aseri et al. 2008) and sunflower (*Helianthus annus* L.) (Akbari et al. 2009). On cannabis, Conant et al. (2017) and Da Cunha Leme Filho et al. (2020a, b) reported greater cannabis height due to biostimulant applications under indoor conditions.

The fact that cannabis height responded differently to the biostmulants application in two growing seasons and different locations might be due the plant stress intensity throughout the seasons. This is not uncommon event, considering that two studies addressing the use of biostimulants on corn (*Zea mays* L.) also found contrasting plant height response depending on the growing season and location (Da Cunha Leme Filho et al. 2021; El-Mekser et al. 2014).

### Chlorophyll content and photosynthetic efficiency

**Fig. 2:**
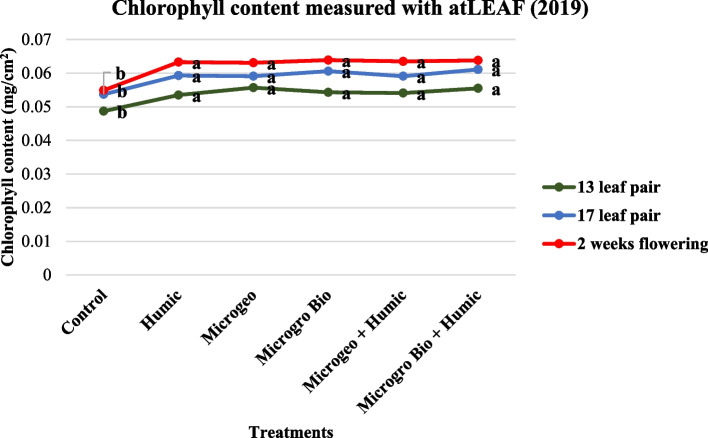
Cannabis chlorophyll content readings collected in 2019 at three growing stages (13, 17, and 2 weeks flowering). Mean within each dot above the treatment followed the same letter by the same growth stage is not significantly different at the 0.05 probability level

**Fig. 3:**
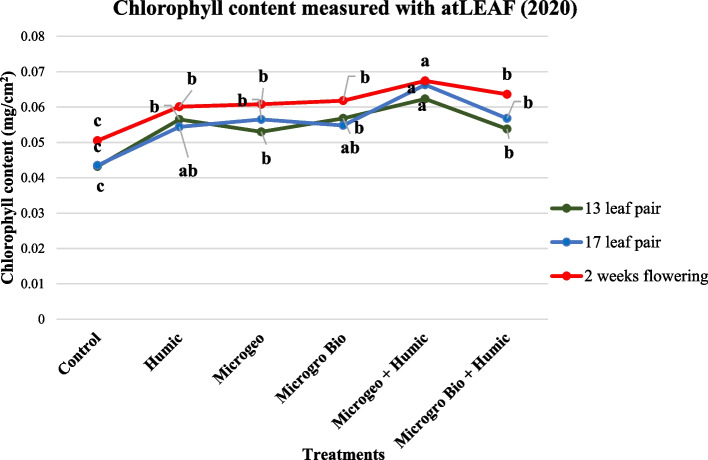
Cannabis chlorophyll content readings collected in 2020 at three growing stages (13, 17, and 2 weeks flowering). Mean within each dot above the treatment followed the same letter by the same growth stage is not significantly different at the 0.05 probability level

**Fig. 4:**
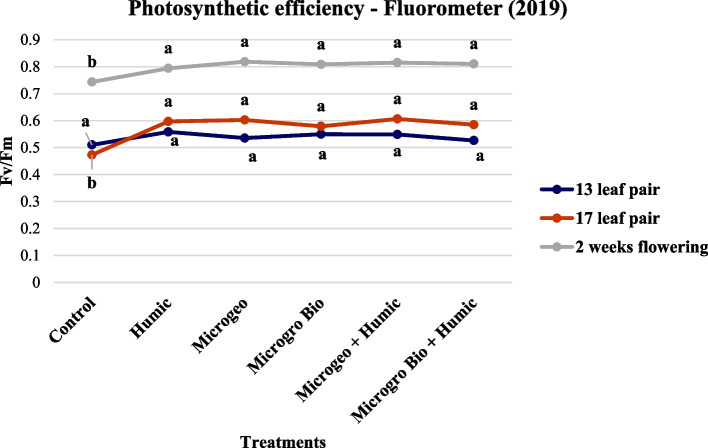
Cannabis photosynthetic efficiency readings collected in 2019 at three growing stages (13, 17, and 2 weeks flowering). Mean within each dot above the treatment followed the same letter by the same growth stage is not significantly different at the 0.05 probability level

**Fig. 5:**
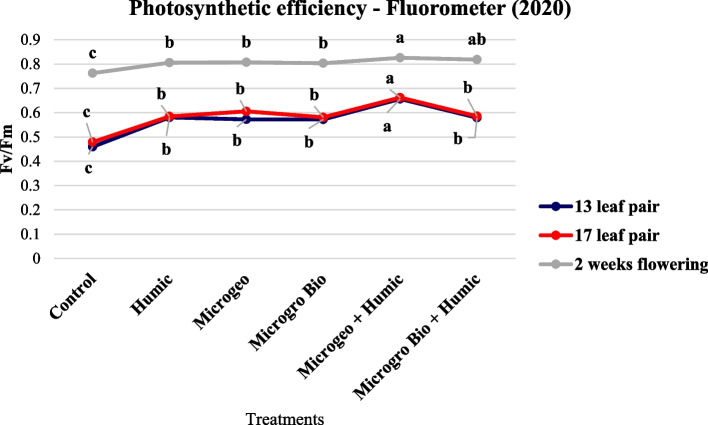
Cannabis photosynthetic efficiency readings collected in 2020 at three growing stages (13, 17, and 2 weeks flowering). Mean within each dot above the treatment followed the same letter by the same growth stage is not significantly different at the 0.05 probability level

Chlorophyll content and photosynthetic efficiency can be indicators of plant stress, however this not always the case. Based on similar response trends we report both measurements in the same section (Figs. 2, 3, 4, and 5). Previous studies have shown a strong relationship between between photosynthetic efficiency and total chlorophyll content (Hazrati et al. 2016; Khaleghi et al. 2012; Sharma et al. 2015). According to Basra (1997), the reduction of chlorophyll pigments will prejudice the photosynthetic mechanisms and consequently lower efficiency.

In 2019, the chlorophyll content and photosynthetic efficiency presented very similar trends, where almost all biostimulant treatments produced numerically higher values than the control at the three data collection periods (Figs. 2 and 4). However, no statistical differences were detected among biostimulant treatments. In 2020, both plant chlorophyll content and photosynthetic efficiency again performed similarly at each of the three measurements. The control values were lower than the rest of the treatments. When comparing among the biostimulant treatments the Microgeo + Humic had the highest values in most of the cases (Figs. 2 and 5). The second season (2020) had higher contrast between control and biostimulants and among the biostimulant treatments. The non-optimal plating date linked to limited vegetative growth in 2020 could be an explanation for this discrepancy as cannabis plants might have faced more stress due certain environmental conditions such as shorter days. Vargas-Hernandez et al. (2017) stated that biostimulants can enhance plant performance or induce plant tolerance to biotic and abiotic stresses, thus biostimulants were more valuable in the second growing season as the plants were potentially under stress. The application of HA increased chlorophyll content on common bean (*Phaseolus vulgaris* L.) under salinity stress (Meganid et al. 2015) and photosynthetic efficiency on rapeseed (*Brassica napus* L.) plants growing under moderate and severe water limitation (Lotfi et al. 2018). Biofertilizers can also affect the level of those plant parameters, mainly when plants are not in optimal conditions (Giri and Mukerji 2004). Two different categories of biofertilizers enhanced chlorophyll density on triticale (× *Triticosecale*) (Younes et al. 2016) and auri (*Acacia auriculiformis*) (Giri et al. 2003) when comparing to control under salinity stress. The photosynthetic efficiency and drought tolerance were also positively affected by the use of biofertilizers on date palm (*Phoenix dactylifera* L.) (Anli et al. 2020). Therefore, similarly to these previous studies, the less favorable conditions of 2020 could have maximized the effects of the biostimulants on chlorophyll content and photosynthetic efficiency of cannabis.

In 2020, Microgeo + Humic had greater chlorophyll content and photosynthetic efficiency than other treatments (Figs. 3 and 5). This aligned to our hypothesis that the integration of both compounds would maximize cannabis performance, however only one of two treatments receiving biofertilizer + HA presented statistically higher values. According to Abou-Aly and Mady (2009), the complementary application of biofertilizer + HA remarkably increase nutrient uptake, total carbohydrates, and thereby photosynthetic pigments on wheat. The integrated application of biofertilizer and HA also increase chlorophyll levels on safflower (*Carthamus tinctorius* L.) (Yadollahi et al. 2015) and basil (*Ocimum basilicum* var. thyrsiflorum) (Boveiri Dehsheikh et al. 2017).

### Greenness and vigor

**Fig. 6:**
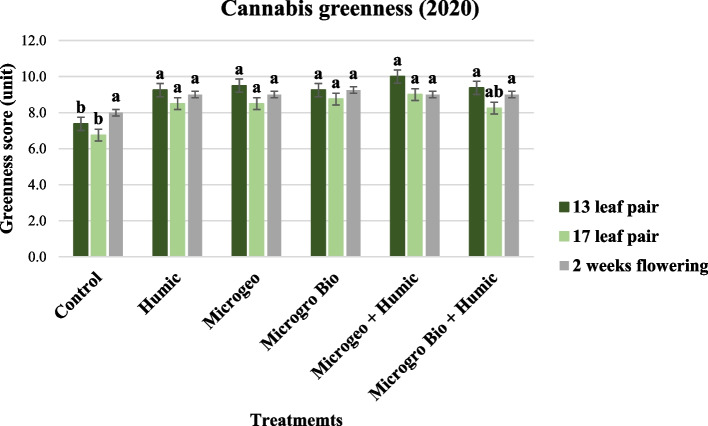
Cannabis greenness score collected in 2020 at three growing stages (13, 17, and 2 weeks flowering). Mean within a column followed the same letter by the same growth stage is not significantly different at the 0.05 probability level

**Fig. 7:**
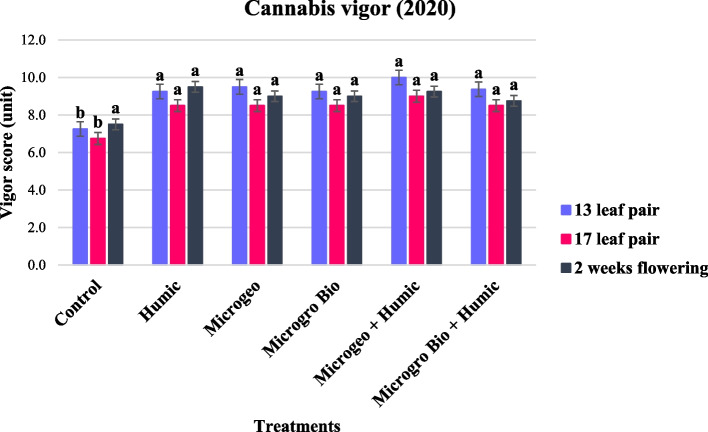
Cannabis vigor score collected in 2020 at three growing stages (13, 17, and 2 weeks flowering). Mean within a column followed the same letter by the same growth stage is not significantly different at the 0.05 probability level

Scores of greenness and vigor were lower for the control that any treatment at the first two measurement times in the 2020 growing season. Generally, greenness and vigor in the last data period (2 weeks flowering) were similar throughout all the treatments and the values collected at 13 and 17 leaf pair showed the control lower than the biostimulant treatments (Figs. 6 and 7). The late planting date occurred in the 2020 growing season exposed the cannabis plants to less favorable conditions to achieve high plant quality scores, thus the biostimulants might had more possibilities to show their effects on mitigating plant stress when comparing against control. Two comprehensive reviews addressing fruits and horticultural crops confirmed the action of biostimulants not only on stress attenuation, but also improvements in appearance, chemical and physical attributes (Drobek et al. 2019; Rodrigues et al. 2020).

### Aboveground biomass

Average aboveground biomass from all treatments in 2019 was 2673 kg ha^−1^ while the average in 2020 was 936 kg ha^−1^. Therefore, the delay of 1 month in the planting could be the cause of 65% decrease in cannabis biomass due to vegetative growth limitation. According to Cazenave et al. (2019), twenty cannabis varieties were sensitive to three planting dates in Virginia and the earliest planting date generally presented the highest biomass, explained by greater branching.

**Fig. 8:**
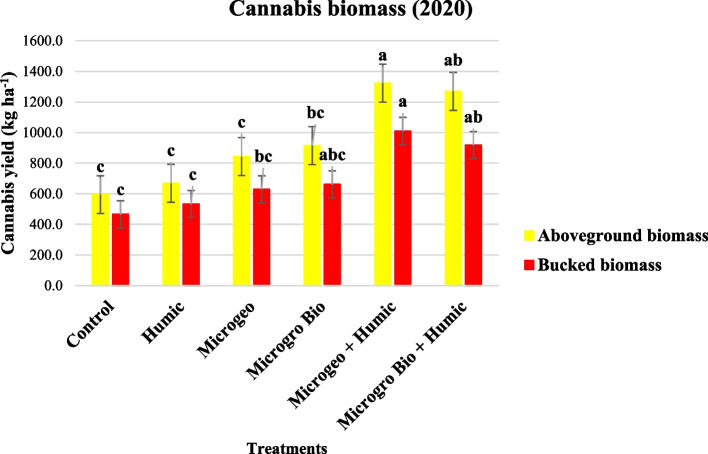
Cannabis aboveground and bucked biomass collected in 2020 at three growing stages (13, 17, and 2 weeks flowering). Mean within a column followed the same letter by the same growth stage is not significantly different at the 0.05 probability level

Similar to cannabis height and greenness and vigor, aboveground biomass and bucked biomass were only different due to treatments in 2020 (Fig. 8). Both treatments receiving the combination of biofertilizer + HA products produced more biomass than the control and the treatments receiving only one biofertilizer or HA. The Microgeo + Humic treatment generally resulted in the highest aboveground and bucked biomass. These biomass results closely approximate to our hypothesis, where the combined use of biofertilizer and HA products would maximize the overall plant performance. However, taking into consideration all the plant parameters evaluated in this study, the synergy of biofertilizers and HA did not have a clean-cut effect as the alone and combined application showed efficacy in different magnitudes throughout the experiment. A study addressing bell and chili pepper (*Capsicum annuum*) under progressive soil salinity gradient presented similar inconsistencies in terms of the individual and integrated application of biofertilizer and HA depending on the plant parameter analyzed (Bacilio et al. 2016). Olivares et al. (2015) tested the application of biofertilizers and HA products in tomato (*Solanum lycopersicum* L.) at seedling (greenhouse) and field condition, which was comparable to this study where the cannabis transplants/plants received the treatments via drench and foliar application. Then, the integrated use of biofertilizer and HA increased tomato seedling shoot dry matter (greenhouse) and leave area (field) in parallel to what occurred with cannabis biomass in this study. Furthermore, fresh and dry corn biomass were significantly increase by the foliar application of biofertilizer + HA (Canellas et al. 2015). The interaction of HA with the development of plants and microbes (biofertilizers) is related to various functional groups as enzyme and hormonal activity (Nardi et al. 2016), plasma membrane permeability (Canellas and Olivares 2014) and photosynthesis and respiration (Nardi et al. 2002).

### Soil CO_2_ evolution

**Fig. 9:**
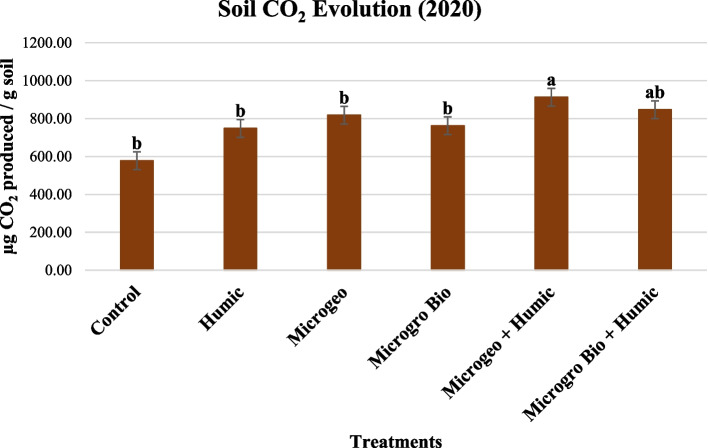
Soil CO_2_ evolution analyzed in the samples collected after harvest in 2020. Mean within a column followed the same letter by the same growth stage is not significantly different at the 0.1 probability level

The results of 2019 growing season did not present statistically significant differences. In 2020, the soil CO_2_ evolution results progressively increased as biofertililzers, HA and the combination of both resources were added in the treatments (Fig. 9). However, only Microgeo + Humic produced soil CO_2_ evolution values greater than the control and the independent application of each biostimulant, but was not different from Microgro Bio + Humic.

Once again, biofertilizers and HA applied in combination presented the highest value but the synergy evidence were not thoroughly clear when analyzing all the treatments. The control did not show statistically significant differences when comparing to biofertilizers/HA applied alone and one of the treatments combining the two resources. Literature has shown more straightforward results of the synergy between biofertilizer and HA on soil microbial activity, particularly in adverse conditions as dry, heat and high salt (Abdelrahman et al. 2021; Aswathy et al. 2017). The soil CO_2_ evolution increased significantly when biofertilizer was applied with two HA doses, and the treatments with the highest HA concentration presented the greatest CO_2_ evolution against lower and no HA addition (El-Sayed and El-Sayed 2020). As soil CO_2_ evolution is directly related to soil microbial activity (Frankenberger Jr and Dick 1983), the application of HA positively affects microbial growth due enzymatic function (Visser 1985) and carbon source (Flaig 1964). A review evaluating the effects of HA on microbial activity indicated that HA dose and bacteria and fungi species have an important influence on whether or not the activity measurement will be increased (Da Cunha Leme Filho et al. 2020a, b).

## Conclusion

The combined and individual use of biofertilizers and HA affected cannabis biochemical and physiological parameters as height, chlorophyll content, photosynthetic efficiency, greenness, vigor, aboveground biomass, and bucked biomass mainly when the growing conditions were not optimal as occurred in 2020 when the plants had fewer days of vegetative growth. In 2019, when the crop was planted in the optimum window, chlorophyll content and photosynthetic efficiency were the only parameters influenced by the application of biostimulants. The discrepancies among the two growing seasons reinforce the evidence of other studies that biostmulant efficacy is maximized under stress conditions. Hawaiian Haze is a photoperiod sensitive variety, and the earlier flowering stimulation could harm the plants as the vegetative growth should be prolonged. However, we recognize the shortfalls of not having unstressed treatments in order to precisely assess and compare how much stress were endured by the cannabis plants in each growing season. Regardless the potential stress exposure that any crop might endure under field conditions, those biostimulants could be an option to improve cannabis yield and quality, as currently, few products are authorized to be applied and the grower have limited alternatives. Moreover, growers might take advantage of biofertilizers and HA as a preventive tool against biotic and abiotic elements. The data gathered in this study could not conclusively confirm that the combined use of biofertilizer + HA is a better practice than individual application considering that both methods affected plant parameters in different magnitudes throughout the growing seasons. This is also valid for soil CO2 evolution, because only one among two treatments in the category of biofertilizer + HA showed to be affective, so not a clear evidence of superiority.

## Data Availability

The authors are willing to provide the any type of raw data to support their findings.

## References

[CR1] Abdelrahman HM, Zaghloul R, Hassan EA, El-Zehery H, Salem A (2021). New strains of plant growth-promoting rhizobacteria in combinations with humic acid to enhance squash growth under saline stress. Egypt J Soil Sci.

[CR2] Abou-Aly H, Mady M (2009). Complemented effect of humic acid and biofertilizers on wheat (Triticum aestivum L.) productivity. Ann Agric Sci Moshtohor.

[CR3] Akbari P, Ghalavand A, Modares SAM. Effects of different nutrition systems and biofertilizer (PGPR) on phenology period yield andyield components of sunflower (Helianthus Annuus L.). Electron J Crop Prod. 2009:119–34.

[CR4] Anli M, Baslam M, Tahiri A, Raklami A, Symanczik S, Boutasknit A, Ait-El-Mokhtar M, Ben-Laouane R, Toubali S, Ait Rahou Y, Ait Chitt M, Oufdou K, Mitsui T, Hafidi M, Meddich A (2020). Biofertilizers as strategies to improve photosynthetic apparatus, growth, and drought stress tolerance in the date palm. Front Plant Sci.

[CR5] Aseri GK, Jain N, Panwar J, Rao AV, Meghwal PR (2008). Biofertilizers improve plant growth, fruit yield, nutrition, metabolism and rhizosphere enzyme activities of Pomegranate (Punica granatum L.) in Indian Thar Desert. Sci Hort.

[CR6] Aswathy T, Johny J, Dhanya M, Sathyan T, Preethy T, Murugan M (2017). Effect of biofertilizers and organic supplements on general and beneficial microbial population in the rhizosphere of black pepper cuttings (Piper nigrum L.). Int J Chem Stud.

[CR7] Bacilio M, Moreno M, Bashan Y (2016). Mitigation of negative effects of progressive soil salinity gradients by application of humic acids and inoculation with Pseudomonas stutzeri in a salt-tolerant and a salt-susceptible pepper. Appl Soil Ecol.

[CR8] Basbag S (2008). Effects of humic acid application on yield and quality of cotton (Gossypium hirsutum L.). Asian J Chem.

[CR9] Basra AS, Basra RK. Mechanisms of environmental stress resistance in plants. Routledge; 1997.

[CR10] Bernstein N, Gorelick J, Zerahia R, Koch S (2019). Impact of N, P, K, and humic acid supplementation on the chemical profile of medical cannabis (Cannabis sativa L). Front Plant Sci.

[CR11] Bevan L, Jones M, Zheng Y. Optimisation of nitrogen, phosphorus, and potassium for soilless production of Cannabis sativa in the flowering stage using response surface analysis. Front Plant Sci. 2021:2587. 10.3389/fpls.2021.764103. 10.3389/fpls.2021.764103 PMC8635921 34868163

[CR12] Boveiri Dehsheikh A, Mahmoodi Sourestani M, Zolfaghari M, Enayatizamir N (2017). The effect of plant growth promoting rhizobacteria, chemical fertilizer and humic acid on morpho-physiological characteristics of basil (Ocimum basilicum var. Thyrsiflorum). J Agric Sci Sustain Prod.

[CR13] Brown P, Saa S. Biostimulants in agriculture. Front Plant Sci. 2015;6. 10.3389/fpls.2015.00671. 10.3389/fpls.2015.00671 PMC4550782 26379695

[CR14] Bulgari R, Franzoni G, Ferrante A (2019). Biostimulants application in horticultural crops under abiotic stress conditions. Agronomy.

[CR15] Butsic V, Brenner JC (2016). Cannabis (Cannabis sativa or C. indica) agriculture and the environment: a systematic, spatially-explicit survey and potential impacts. Environ Res Lett.

[CR16] Callaway JC (2004). Hempseed as a nutritional resource: an overview. Euphytica.

[CR17] Calvo P, Nelson L, Kloepper JW (2014). Agricultural uses of plant biostimulants. Plant Soil.

[CR18] Canellas LP, da Silva SF, Olk DC, Olivares FL (2015). Foliar application of plant growth-promoting bacteria and humic acid increase maize yields. J Food Agric Environ.

[CR19] Canellas LP, Olivares FL (2014). Physiological responses to humic substances as plant growth promoter. Chem Biol Technol Agric.

[CR20] Caplan D, Dixon M, Zheng Y (2017). Optimal rate of organic fertilizer during the vegetative-stage for cannabis grown in two coir-based substrates. HortScience.

[CR21] Cazenave A-B, Davies B, Balota M. Determine the optimal planting date of twenty varieties of hemp (Cannabis sativa L.) for suitable growth and production in Southeastern Virginia. 2019.

[CR22] Chen Y, Aviad T, MacCarthy P, Clapp CE, Malcolm RL, Bloom PR (1990). Effects of humic substances on plant growth 1. Humic substances in soil and crop sciences: selected readings.

[CR23] Cherney JH, Small E (2016). Industrial hemp in North America: production, politics and potential. Agronomy.

[CR24] Conant R, Walsh R, Walsh M, Bell C, Wallenstein M (2017). Effects of a microbial biostimulant, Mammoth PTM, on Cannabis sativa bud yield. J Hortic.

[CR25] Da Cunha Leme Filho JF, Thomason WE, Evanylo GK, Zhang X, Strickland MS, Chim BK, Diatta AA (2020). Biochemical and physiological responses of Cannabis sativa to an integrated plant nutrition system. Agron J.

[CR26] Da Cunha Leme Filho JF, Thomason WE, Evanylo GK, Zhang X, Strickland MS, Chim BK, Diatta AA (2020). The synergistic effects of humic substances and biofertilizers on plant development and microbial activity: a review. Int J Plant Soil Sci.

[CR27] Da Cunha Leme Filho JF, Thomason WE, Evanylo GK, Zhang X, Strickland MS, Chim BK, Diatta AA (2021). An integrated plant nutrition system (IPNS) for corn in the Mid-atlantic USA. J Plant Nutr.

[CR28] Detyniecki K, Hirsch L (2015). Marijuana use in epilepsy: the myth and the reality. Curr Neurol Neurosci Rep.

[CR29] Drobek M, Frąc M, Cybulska J (2019). Plant biostimulants: importance of the quality and yield of horticultural crops and the improvement of plant tolerance to abiotic stress—a review. Agronomy.

[CR30] du Jardin P (2015). Plant biostimulants: definition, concept, main categories and regulation. Sci Hortic.

[CR31] D’andrea P. Processo de compostagem líquida contínua-CLC e biofertilizante. Microbiol Indústria e Comércio LTDA BR/SP 2099. 2002.

[CR32] Eisenstein M (2015). Medical marijuana: showdown at the cannabis corral. Nature.

[CR33] El-Ghamry AM, El-Hai KA, Ghoneem KM (2009). Amino and humic acids promote growth, yield and disease resistance of faba bean cultivated in clayey soil. Aust J Basic Appl Sci.

[CR34] El-Mekser HKA, Mohamed ZEM, Ali MAM (2014). Influence of humic acid and some micronutrients on yellow corn yield and quality. World Appl Sci J.

[CR35] El-Sayed MAM, El-Sayed MT (2020). Effect of humic acid, biofertilizers and mineral phosphate on soil microbial activity and productivity of pea plants under Toshka conditions. Alexandria Sci Exch J.

[CR36] EL Sabagh A, Islam MS, Hossain A, Iqbal MA, Mubeen M, Waleed M, Reginato M, Battaglia M, Ahmed S, Rehman A (2022). Phytohormones as growth regulators during abiotic stress tolerance in plants. Front Agron.

[CR37] ElSohly M, Gul W (2014). Constituents of cannabis sativa. Handb Cannabis.

[CR38] Fike J (2016). Industrial hemp: renewed opportunities for an ancient crop. Crit Rev Plant Sci.

[CR39] Finnan J, Styles D. Hemp: a more sustainable annual energy crop for climate and energy policy. 2013.

[CR40] Flaig W (1964). Effects of micro-organisms in the transformation of lignin to humic substances. Geochim Cosmochim Acta.

[CR41] Fortenbery TR, Bennett M (2004). Opportunities for commercial hemp production. Appl Economic Perspect Policy.

[CR42] Frankenberger WT, Dick WA (1983). Relationships between enzyme activities and microbial growth and activity indices in soil. Soil Sci Soc Am J.

[CR43] Franzluebbers AJ (2016). Should soil testingservices measure soil biological activity?. Agric Environ Lett.

[CR44] Franzluebbers AJ (2018). Soil-test biological activity with the flush of CO2: III. Corn yield responses to applied nitrogen. Soil Sci Soc Am J.

[CR45] Franzluebbers AJ, Haney RL, Honeycutt CW, Schomberg HH, Hons FM (2000). Flush of carbon dioxide following rewetting of dried soil relates to active organic pools. Soil Sci Soc Am J.

[CR46] Fuentes-Ramirez LE, Caballero-Mellado J, Siddiqui ZA (2006). Bacterial biofertilizers. PGPR: biocontrol and biofertilization.

[CR47] Giri B, Mukerji KG (2004). Mycorrhizal inoculant alleviates salt stress in Sesbania aegyptiaca and Sesbania grandiflora under field conditions: evidence for reduced sodium and improved magnesium uptake. Mycorrhiza.

[CR48] Giri B, Kapoor R, Mukerji KG (2003). Influence of arbuscular mycorrhizal fungi and salinity on growth, biomass, and mineral nutrition of Acacia auriculiformis. Biol Fertil Soils.

[CR49] Gorelick J, Bernstein N, Chandra S, Lata H, ElSohly MA (2017). Chemical and physical elicitation for enhanced cannabinoid production in cannabis. Cannabis sativa L. - botany and biotechnology.

[CR50] Gryndler M, Hršelová H, Sudová R, Gryndlerová H, Řezáčová V, Merhautová V (2005). Hyphal growth and mycorrhiza formation by the arbuscular mycorrhizal fungus Glomus claroideum BEG 23 is stimulated by humic substances. Mycorrhiza.

[CR51] Hagerty SL, Williams SLY, Mittal VA, Hutchison KE (2015). The cannabis conundrum: thinking outside the THC box. J Clin Pharmacol.

[CR52] Haney RL, Brinton WF, Evans E (2008). Soil CO_2_ respiration: comparison of chemical titration, CO_2_ IRGA analysis and the Solvita gel system. Renew Agric Food Syst.

[CR53] Hanuš LO, Meyer SM, Muñoz E, Taglialatela-Scafati O, Appendino G (2016). Phytocannabinoids: a unified critical inventory. Nat Prod Rep.

[CR54] Hazrati S, Tahmasebi-Sarvestani Z, Modarres-Sanavy SAM, Mokhtassi-Bidgoli A, Nicola S (2016). Effects of water stress and light intensity on chlorophyll fluorescence parameters and pigments of Aloe vera L. Plant Physiol Biochem.

[CR55] Jensen B, Chen J, Furnish T, Wallace M (2015). Medical marijuana and chronic pain: a review of basic science and clinical evidence. Curr Pain Headache Rep.

[CR56] Kaiser C, Cassady C, Ernst M. Industrial hemp production. Center for Crop Diversification, University of Kentucky; 2015.

[CR57] Kauffman GL, Kneivel DP, Watschke TL (2007). Effects of a biostimulant on the heat tolerance associated with photosynthetic capacity, membrane thermostability, and polyphenol production of perennial ryegrass. Crop Sci.

[CR58] Khaleghi E, Arzani K, Moallemi N, Barzegar M (2012). Evaluation of chlorophyll content and chlorophyll fluorescence parameters and relationships between chlorophyll a, b and chlorophyll content index under water stress in Olea europaea cv. Dezful. World Acad Sci Eng Technol.

[CR59] Laila FH, Shahin M, Mahdy H, Amira K, Hassan H (2015). Beneficial effect of NPK, pigeon manure tea and microbial fertilizers as soil application on growth of Toffahi and Picual olive seedlings. J Agric Technol.

[CR60] Lotfi R, Kalaji HM, Valizadeh GR, Khalilvand Behrozyar E, Hemati A, Gharavi-Kochebagh P, Ghassemi A (2018). Effects of humic acid on photosynthetic efficiency of rapeseed plants growing under different watering conditions. Photosynthetica.

[CR61] Lyu D, Backer R, Robinson WG, Smith DL (2019). Plant growth-promoting rhizobacteria forcannabis production: yield, cannabinoid profile and disease resistance. Front Microbiol.

[CR62] Mabood F, Zhou X, Smith DL (2014). Microbial signaling and plant growth promotion. Can J Plant Sci.

[CR63] Mediavilla V, Jonquera M, Schmid-Slembrouck I, Soldati A (1998). Decimal code for growth stages of hemp (Cannabis sativa L.). J Int Hemp Assoc.

[CR64] Meganid AS, Al-Zahrani HS, El-Metwally M (2015). Effect of humic acid application on growth and chlorophyll contents of common bean plants (Phaseolus vulgaris L.) under salinity stress conditions. Int J Innov Res Sci Eng Technol.

[CR65] Moreno-Salazar R, Sánchez-García I, Chan-Cupul W, Ruiz-Sánchez E, Hernández-Ortega HA, Pineda-Lucatero J, Figueroa-Chávez D (2020). Plant growth, foliar nutritional content and fruit yield of Capsicum chinense biofertilized with Purpureocillium lilacinum under greenhouse conditions. Sci Hortic.

[CR66] Naher UA, Panhwar QA, Othman R, Ismail MR, Berahim Z (2016). Biofertilizer as a supplement of chemical fertilizer for yield maximization of rice. J Agric Food Dev.

[CR67] Nardi S, Pizzeghello D, Muscolo A, Vianello A (2002). Physiological effects of humic substances on higher plants. Soil Biol Biochem.

[CR68] Nardi S, Pizzeghello D, Schiavon M, Ertani A (2016). Plant biostimulants: physiological responses induced by protein hydrolyzed-based products and humic substances in plant metabolism. Sci Agric.

[CR69] O’Dell CR, Ramsey P, White RC, Maxey F (1989). String weave fresh market tomatoes: summer and fall production guide.

[CR70] Olivares FL, Aguiar NO, Rosa RCC, Canellas LP (2015). Substrate biofortification in combination with foliar sprays of plant growth promoting bacteria and humic substances boosts production of organic tomatoes. Sci Hortic.

[CR71] Peña-Méndez EM, Havel J, Patočka J (2005). Humic substances–compounds of still unknown structure: applications in agriculture, industry, environment, and biomedicine. J Appl Biomed.

[CR72] Puglisi E, Fragoulis G, Ricciuti P, Cappa F, Spaccini R, Piccolo A, Trevisan M, Crecchio C (2009). Effects of a humic acid and its size-fractions on the bacterial community of soil rhizosphere under maize (Zea mays L.). Chemosphere.

[CR73] Rodrigues M, Baptistella JLC, Horz DC, Bortolato LM, Mazzafera P (2020). Organic plant biostimulants and fruit quality—a review. Agronomy.

[CR74] Romero AM, Vega D, Correa OS (2014). Azospirillum brasilense mitigates water stress imposed by a vascular disease by increasing xylem vessel area and stem hydraulic conductivity in tomato. Appl Soil Ecol.

[CR75] Ronga D, Caradonia F, Setti L, Hagassou D, Giaretta Azevedo C, Milc J, Pedrazzi S, Allesina G, Arru L, Francia E. Effects of innovative biofertilizers on yield of processing tomato cultivated in organic cropping systems in northern Italy. 2018.

[CR76] Saloner A, Bernstein N (2020). Response of medical cannabis (Cannabis sativa L.) to nitrogen supply under long photoperiod. Front Plant Sci.

[CR77] Saloner A, Bernstein N (2021). Nitrogen supply affects cannabinoid and terpenoid profile in medical cannabis (Cannabis sativa L.). Ind Crops Prod.

[CR78] Saloner A, Bernstein N (2022). Effect of potassium (K) supply on cannabinoids, terpenoids and plant function in medical cannabis. Agronomy.

[CR79] Sani B (2014). Foliar application of humic acid on plant height in canola. APCBEE Procedia.

[CR80] Sharma DK, Andersen SB, Ottosen CO, Rosenqvist E (2015). Wheat cultivars selected for high Fv/Fm under heat stress maintain high photosynthesis, total chlorophyll, stomatal conductance, transpiration and dry matter. Physiol Plant.

[CR81] Shiponi S, Bernstein N (2021). The highs and lows of P supply in medical cannabis: effects on cannabinoids, the ionome, and morpho-physiology. Front Plant Sci.

[CR82] Singh M, Dotaniya ML, Mishra A, Dotaniya CK, Regar KL, Lata M, Bisht JK, Meena VS, Mishra PK, Pattanayak A (2016). Role of biofertilizers in conservation agriculture. Conservation agriculture: an approach to combat climate change in Indian Himalaya.

[CR83] Small E (2016). Cannabis: a complete guide.

[CR84] Small E, Cronquist A. A practical and natural taxonomy for cannabis. Taxon. 1976:405–435.

[CR85] Tahir MM, Khurshid M, Khan MZ, Abbasi MK, Kazmi MH (2011). Lignite-derived humic acid effect on growth of wheat plants in different soils. Pedosphere.

[CR86] Tattini M, Bertoni P, Landi A, Traversi M. Effect of humis acids on growth and biomass partitioning of conteiner-grown olive plants. 1990.

[CR87] Turner JC, Hemphill JK, Mahlberg PG (1978). Quantitative determination of cannabinoids in individual glandular trichomes of Cannabis sativa L.(Cannabaceae). Am J Bot.

[CR88] Ullah A, Manghwar H, Shaban M, Khan AH, Akbar A, Ali U, Ali E, Fahad S (2018). Phytohormones enhanced drought tolerance in plants: a coping strategy. Environ Sci Pollut Res.

[CR89] Ulukan H (2008). Effect of soil applied humic acid at different sowing times on some yield components in wheat (Triticum spp.) hybrids. Int J Bot.

[CR90] Vargas-Hernandez M, Macias-Bobadilla I, Guevara-Gonzalez RG, Romero-Gomez SdJ, Rico-Garcia E, Ocampo-Velazquez RV, Alvarez-Arquieta LdL, Torres-Pacheco I. Plant hormesis management with biostimulants of biotic origin in agriculture. Front Plant Sci. 2017;8. 10.3389/fpls.2017.01762. 10.3389/fpls.2017.01762 PMC5645530 29081787

[CR91] Visser SA (1985). Effect of humic acids on numbers and activities of micro-organisms within physiological groups. Org Geochem.

[CR92] Yadollahi P, Asgharipour MR, Kheiri N, Ghaderi A (2015). Effects of drought stress and different types of organic fertilizers on the yield and yield components of safflower (Carthamus tinctorius L.).

[CR93] Younes KA, Raouf SS, Mohammad S, Morteza B. Effect of zinc and bio fertilizers on antioxidant enzymes activity, chlorophyll content, soluble sugars and proline in triticale under salinity condition. Not Bot Horti Agrobot Cluj-Napoca. 2016;44. 10.15835/nbha44110224.

[CR94] Zuardi AW (2006). History of cannabis as a medicine: a review. Braz J Psychiatry.

